# Observations on the Tread-Mill

**Published:** 1823-08

**Authors:** Benj. Hutchinson

**Affiliations:** Fellow of the Royal College of Surgeons. Southwell


ART. V.—*Observations on the Tread-Mill.* By BENJ. HUTCHINSON, Esq. Addressed to the Rev. JOHN THOMAS BECHIN, Prebendary of the Collegiate Church of Southwell, and WILLIAM WYLDE, Esq. Visiting Justices of the Nottinghamshire House of Correction, at Southwell.

GENTLEMEN,—Having had the honour of holding the important and responsible situation of surgeon to the Nottinghamshire House of Correction at Southwell, during the last twenty-eight years, I cannot but feel peculiarly interested in every the most minute circumstance connected with the improvement and preservation of the health of the unfortunate inhabitants of that excellently-managed prison.

Influenced by these impressions, I have the honour of addressing to you such observations as have been elicited by a recent perusal of Sir John Cox Hippisley's "Supplementary

Note on the Use of the Tread-Mill in Prisons;" and I trust you will allow me to add, that these few remarks are submitted to your perusal with every delicacy of feeling, resulting from the well-known and highly respectable character of the author of this " Supplementary Note."

I believe the axiom is universally admitted, that bodily exercise is essentially requisite to the preservation and improvement of health, so long as the bounds of moderation are not encroached upon. It is also equally manifest, that exercise taken too violently is attended with the same disadvantages as a total want of it. On this subject I am convinced that I need not fear any opposition of opinion, and it will therefore be unavailing to dwell longer on its discussion.

The first objection, of a medical character, to the use of the tread-wheel, in the " Supplementary Note" above alluded to, states that, in the Coldbath-fields' prison, there was scarcely an individual of the group who did not complain of pain in the back of their legs, in their shoulders, thighs, and in the parts in conjunction with the groins. I freely admit that the operation of the wheel is productive of certain muscular pains, the natural effect of considerable muscular exertion; not, however, to an extent exceeding that which, I imagine, is not only highly salutary, but in every respect truly desirable. As to the accident on the machinery, by which the boys at work on another wheel in the same prison had their feet miserably crushed, I can only assert that accidents of this description have not happened in the Nottinghamshire House of Correction; neither do I see any probability of their occurrence, excepting by the extreme carelessness of those on the wheel.

I confess myself happy in this opportunity of adding my feeble testimony to the justly-merited eulogiums contained in Sir John Cox Hippisley's " Supplementary Note," on the talents and erudition of Dr. Mason Good, with whose professional writings I have been long and well acquainted, and from which I have derived very important information: they fully entitle him to the grateful feeling of his brethren, and to the literary credit so fairly and honourably awarded him. It cannot, therefore, be otherwise than a matter of regret to be compelled to differ essentially from his opinion on a subject, neither admitting nor embracing any speculative or theoretical argument, but depending solely on accuracy of statement and attentive observation. I shall therefore, I trust, Gentlemen, stand excused for thus noticing what I conceive to be the erroneous opinions of Dr. Good on the much-agitated topic of the use of the tread-wheel, as an instrument of punishment in our prisons.

Dr. Good agrees in opinion with the gentlemen composing the Committee for Prison Discipline, that the tread-mill is an object of peculiar terror. I also am of opinion with Dr. Good, that the objects incurring this punishment have a dread of its fatiguing labour, probably in the exact proportion that was wished and expected by those who suggested this highly beneficial mode of obviating the repetition of crime. One among the multitude of Dr. Good's objections to the use of this mill is "the tortuous and irksome attitude of treading upon the TOES up an endless and nearly-perpendicular hill; the heels, which should chiefly bear the weight of the body, rendered useless; the natural line of gravity dislocated; the hands forced into a rigid and benumbing grasp; and the extensor muscles of the legs for ever on a painful, and, necessarily therefore, on a mischievous and morbid stretch." My answer to these strong observations of Dr. Good is the result of an accurate and most attentive inspection of the effects produced by the exercise of the tread-mill: I must therefore be excused in offering an opinion of a diametrically opposite nature. I am compelled to declare, that the attitude is neither "tortuous" nor "irksome," and that, by an examination of the prisoners on the tread-mill, it will uniformly be observed that the weight of the body by no means rests on the toes, excepting by the will of the prisoner; but that, on the contrary, nearly two-thirds of the length of the foot are engaged in performing this exercise of ascent,—very essentially relieving, therefore, the muscular and tendinous exertion and extension which would be requisite, were the toes only made the points of the superincumbent weight: and, indeed, I am fully prepared to state that exercise, conducted in the manner pointed out by Dr. Good, could be continued but a very short time, and would be productive of never-ending lameness and misery to the prisoner who had suffered this torture. A very great relief is also offered to the experienced mill-treader, (an experience fully obtained by the practice of a very few hours,) in his ability to change at pleasure his position on the wheel, to a lateral, dorsal, and semi-dorsal position. In the lateral position of his body, he is enabled to place the whole length of his feet on the stepping-boards, thereby affording very great relief from fatigue to the various large muscles and tendons of the thighs, legs, and feet, and securing him from any severe spasmodic contraction of those parts, the probable consequence of long-continued exertion. The natural line of gravity, during the operation of the tread-mill, is by no means dislocated: in many instances, indeed, I have observed that it has not been in the slightest degree interrupted, the body preserving the same line as in any other act of locomotion. It has never occurred to me to hear any complaint of the hands being forced into a rigid and benumbing grasp: neither, from the degree of force requisite to support the body, can I imagine that any such effects can ever be produced. My experience has also taught me that the extensor muscles of the legs have never sustained any "mischievous and morbid stretch;" and I must beg permission to declare, that, from a long and attentive consideration of the effects of muscular motion, it is impossible to conceive that any such inconveniences are at all likely to be the result. That the tread-wheel is an instrument of peculiar terror, cannot but be considered an argument of the most forcible kind in its favour. So long as punishments excite no dread, their efficiency in the prevention of crime cannot be otherwise than comparatively trifling and nugatory. The concluding observations of Dr. Good, in Sir John Cox Hippisley's "Supplementary Note," are followed by a letter from one of his distinguished medical friends, senior physician to the King, and formerly a physician in the navy. This gentleman, Sir Gilbert Blane, is in no respect adverse to the use of the tread-wheel; he merely thinks that there can be no necessity for making this the exclusive mode of punishing the inmates of our prisons: he appears to imagine that the tread-wheel might operate as a severe punishment for offences of a more aggravated nature, and the hand-crank, or winch machinery, for those of minor atrocity.

The surgeon of the prison at Shepton Mallet entertains some fears that the operation of the tread-mill may render the prisoners unable to work at any other labour for some time after his discharge from prison, from the effects produced not only upon his arms, but upon the principal muscles of the body. After the most attentive examination of the prisoners who have laboured at the tread-mill, I shall deem myself fully authorised to assert that no such pernicious effect need to be apprehended. The extension of the muscles of the arms is neither so great nor so long continued, as to produce any morbid or deranged action in these important parts of the human frame. These remarks are the result of accurate observation, and not elicited by any preconceived theory, or predilection for the instrument of punishment under consideration.

With your permission, I will now, Gentlemen, beg leave to make a remark on the extract of a letter from the chairman of the Quarter Sessions of the county of Surrey, dated the 30th of December, 1822, intimating that he had made inquiry of the governor of the House of Correction at Brixton, whether he ever knew, or heard from any of the prisoners under his care, that the labour of the tread-mill had affected their limbs or muscles in any way so as to be injurious to them? That his reply was prompt and distinct: he said he never heard of such a complaint, nor indeed of any complaint of the kind, from any of them; and that a woman, who went to work with a rheumatic complaint, had declared that her rheumatism was completely cured. On this particular case I have to remark, that a man of the name of Pearson, a prisoner in the Nottinghamshire House of Correction, made a complaint to me, about ten days past, of suffering most severely from chronic rheumatism in one of his lower limbs, accompanied with some tumefaction of the joints, and indeed of the whole structure of this limb. Among other means of relief proposed, I told him that he should be excused from the labour of the tread-mill a few days; and I was giving the necessary directions to the turnkey, when the man particularly requested that he might be allowed to continue his exercise on the wheel, as his pains were very considerably alleviated during this movement of the limb; and he has regularly continued it with manifest advantage.

I perfectly concur in opinion with the surgeon of the Brixton House of Correction, that varicose veins of the lower extremities are much more likely to be prevented than produced by exercise of this description. That hernia, or other injury to the bodies of the prisoners, cannot be the consequence of the tread-mill exercise, more frequently than labour of any other description, is equally clear; the muscles and other parts of the human frame, the seats of these accidents, sustaining no violence, and no unnatural action of any description.

Dr. Good again, in an extract of a letter dated January 17th, 1823, says, that Mr. ——, the surgeon of the Brixton House of Correction, will never advise the discipline he has recommended, that of walking on *tiptoe* up high and almost perpendicular hills to any patient of his in his private practice. I shall again take the liberty of repeating that, had Dr. Good attentively examined the real state of the mode of stepping on the tread-mill, he would immediately have discerned that what I have before advanced is the plain and positive fact, that two-thirds of the foot is engaged in performing this labour of ascent; that walking on *tiptoe* up high and almost perpendicular hills, is not the exercise produced by this instrument; and that accidents, of any description, can be the effect only of the extreme of carelessness and inattention on the part of the prisoners. On this subject, the opinions of Sir Gilbert Blane, Sir William Blizard, and of Dr. Good, are entitled to, and will doubtless obtain, that share of attention and respect to which their talents and characters confer so just and well-merited a claim. My own sentiments and assertions are the result of actual observation, and of close and diligent inquiry during my professional attendance and duties on the sick prisoners of the Nottinghamshire House of Correction. They are respectfully offered, Gentlemen, to your consideration, under the assurance of your interest in every matter connected with the discipline and the health of these unfortunate offenders against the laws of their country.

I believe it to be the almost unanimous opinion of the medical officers of the navy, with many of the most respectable of whom I have had frequent opportunities of conversing, that seamen are not more subject to varicose affections of the legs, or to herniæ, than any other class of people accustomed to the laborious exercise of their muscles; and the fair and very impartial statement of Sir John Cox Hippisley on this particular point appears in perfect unison with my own observations.

The copies of the communications made to the Secretary of State for the Home Department, respecting the use of tread-wheels in gaols or houses of correction, which were ordered by the House of Commons to be printed in March 1823, consist of communications from twenty different counties, in which the use of the tread-wheel had been then adopted. The result of this mass of most respectable evidence speaks loudly in favour of the highly salutary and safe operation of this mode of preventing a repetition of crime. It very clearly points out the absence of any the most insignificant accidents, but what were the effects of carelessness on the part of the prisoners; and that this species of labour tends to the preservation and improvement of the health of the prisoners, rather than in any respect to injure it, either by inducing herniæ, varicose swellings of the legs, pectoral diseases, or internal affections of any denomination.

In a statement ordered to be printed by the House of Commons, the 2d of May, 1823, entitled "Further Papers relating to the Penitentiary at Millbank," Mr. Copland Hutchison gives the following testimony in favour of the operation of the tread-mill:—"I consider it my duty, therefore, to make this statement to the committee, and to refer them to my Quarterly Report, dated October 4th last, where it will be found that I have adverted to this subject, and also in communications to the committee of an earlier date. The pump and mill now in operation in the Penitentiary give exercise only to the muscles of the arms and trunk; whereas, such a machine as the tread-mill would give exercise to every voluntary muscle of the body, and, in my opinion, would greatly contribute to the preservation of the health of the prisoners in this establishment."

Allow me, Gentlemen, to conclude with the assurance, that, in giving you the trouble of perusing this letter, I am actuated solely, I trust, by a proper sense of my duty as medical attendant of the Nottinghamshire House of Correction; a part of which duty I believe myself to be discharging by submitting to you my opinion of the salutary operation of the instrument in question. I am fully aware that I am opposing the sentiments and assertions of gentlemen whose talents I must admire, and by whose labours and learning I have been essentially instructed. Yet permit me to repeat, that I trouble you with a detail of facts only; the subject admitting neither of discussion nor argument.

I have the honour of subscribing myself, Gentlemen, your obedient servant,

BENJ. HUTCHINSON,

*Fellow of the Royal College of Surgeons.*

*Southwell; June* 1, 1823.

